# Immunoassay for Colistin Monitoring in Critically Ill Patients Receiving Colistin Methanesulfonate Therapy

**DOI:** 10.3390/ph19060880

**Published:** 2026-06-01

**Authors:** Yury A. Surovoy, Inna A. Galvidis, Akmal I. Alimov, Zhanhui Wang, Artem O. Melekhin, Maksim A. Burkin

**Affiliations:** 1 Department of Critical Care, University College of London Hospital, London NW1 2BU, UK; 2 Faculty of Science and Technology, Middlesex University, London NW4 4BT, UK; 3 Department of Immunology, I. Mechnikov Research Institute for Vaccines and Sera, Moscow 105064, Russia; galvidis@yandex.ru (I.A.G.); aai.alimov@gmail.com (A.I.A.); 4 Faculty of Medicine, M.V. Lomonosov Moscow State University, Moscow 119991, Russia; 5 Federal Center for Treatment and Rehabilitation, Ministry of Health, Moscow 125367, Russia; 6 College of Veterinary Medicine, China Agricultural University, Beijing 100193, China; wangzhanhui@cau.edu.cn; 7 Federal Center for Animal Health, Vladimir 600901, Russia; artem150196@mail.ru

**Keywords:** colistin, polymyxins, therapeutic drug monitoring, ELISA, pharmacokinetics

## Abstract

**Background/Objectives**: Colistin (COL), administered as a prodrug colistimethate sodium (CMS), is commonly used to treat infections caused by multidrug-resistant Gram-negative bacteria in critically ill patients. Given high CMS instability, very complex and variable pharmacokinetics (PK) and high incidence of toxicity, therapeutic drug monitoring (TDM) of active COL might play an important role. This study aimed to develop and validate an accessible immunoassay-based approach for COL monitoring in human serum. **Methods**: A direct competitive enzyme-linked immunosorbent assay (dcELISA) was developed using polyclonal (pAb) anti-polymyxin antibody alongside a polymyxin B–horseradish peroxidase conjugate. CMS conversion to COL along with serum deproteinization was achieved using 5% trichloroacetic acid (TCA) treatment at 37 °C. Assay accuracy and precision were assessed by spike-and-recovery experiments in healthy volunteer serum. The assay was applied to serum samples from critically ill patients with burns or pneumonia receiving CMS therapy. The reliability of the measurements was confirmed by parallel dcELISA based on a reference monoclonal antibody (mAb) against fragmented polymyxin molecule. **Results**: Both ELISA formats demonstrated high sensitivity, with limits of detection of 0.053 ng/mL (pAb) and 0.047 ng/mL (mAb). TCA treatment achieved maximal CMS hydrolysis under tested conditions within one hour. Clinical sample analysis showed excellent agreement between the two assays (R^2^ = 0.996), with Bland–Altman analysis revealing a minimal bias of 3.7%. Exploratory PK analysis in burn patients demonstrated increased total drug volume of distribution (45.7–64.9 L) and clearance (8.3–16.3 L/h). **Conclusions**: This is the first report of ELISA for COL TDM in critically ill patients. The method offers acceptable analytical performance and practical simplicity, with potential to broaden TDM access beyond specialist centers.

## 1. Introduction

Lipopeptide antibiotics, polymyxin B (PMB) and polymyxin E (colistin—COL), were first introduced into clinical practice in the middle of the previous century [1]. Initially their use was limited due to the high incidence of nephrotoxicity and development of safer and better-tolerated alternatives [2]. However, the modern era of global spread of antimicrobial resistance [3] has driven their return into clinical practice [4], and they are increasingly used to treat infections caused by multidrug-resistant Gram-negative bacteria [5]. Polymyxins are cyclical lipopeptides [1], which bind to the bacterial LPS complex, disrupt the cell wall and cause cell lysis [6,7].

**Figure 1:**
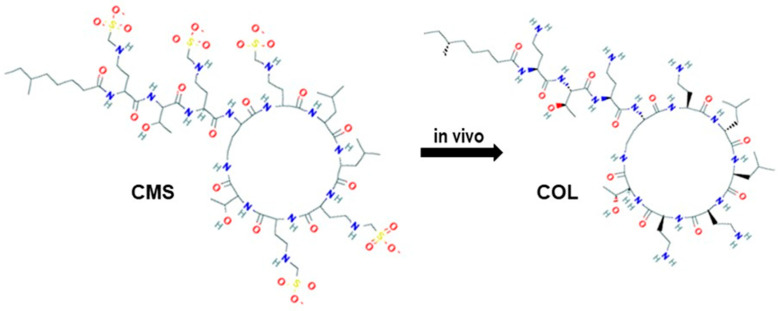
Conversion of CMS into COL via hydrolysis in vivo.

Colistimethate sodium (CMS), a COL prodrug, is a commonly used COL formulation, and in 2024 it was registered for intravenous use in Russia [8]. CMS is derived through formaldehyde-mediated sulfomethylation of the five free primary amino groups of COL [1]. This modification of diaminobutyric acid residues of colistin results in the attachment of 0 to 2 methanesulfonate groups per residue (Figure 1). Consequently, CMS represents a complex mixture of methanesulfonated derivatives, which themselves do not possess any antibacterial activity [4,9].

COL, the initial substrate for CMS production, is isolated from cultures of *Bacillus polymyxa* var. Colistinus and consists of several components, approximately 85% of which are a mixture of colistin A and colistin B, differing by a methyl group in the structure of their N-terminal acyl chains [10,11]. Thus, both COL and CMS are chemically heterogeneous, and the composition of their components in pharmaceutical formulations may vary depending on the manufacturer and batch [10].

CMS is an inactive substance which undergoes hydrolysis in aqueous solutions and in the human body; partially hydrolyzed intermediates ultimately convert to the active antibiotic, COL [11]. Drug elimination is a combination of several mechanisms. In addition to hydrolysis, CMS gets excreted by the kidneys, whereas COL mostly gets reabsorbed by the kidney tubular system and its clearance predominantly depends on non-renal mechanisms. Overall, the concentration of the active drug COL is directly proportional to the CMS dose and the conversion clearance of CMS to COL, and indirectly proportional to CMS renal clearance, as well as COL renal and non-renal clearances [12]. Renal impairment leads to a significant increase in the fraction of CMS which gets converted to COL, putting patients with renal dysfunction at higher risk of toxicity [13,14].

Critically ill patients are one of the main populations where COL is used due to the high prevalence of multidrug-resistant bacteria. COL pharmacokinetics (PK) in this group is very variable owing to the high incidence of organ failure, capillary leak and use of extracorporeal support [15]. Therapeutic drug monitoring (TDM) of COL should be considered in view of difficult-to-predict PK and a narrow therapeutic window [16]. The efficacy of CMS therapy is commonly assessed using the target area under the plasma concentration–time curve over 24 h (AUC_0–24_) between 50 and 100 mg × h/L based on animal efficacy models and nephrotoxicity data [16,17]. This value corresponds to an average steady-state COL plasma concentration (C_ss_) of 2 mg/L [16].

An established approach to CMS quantification in plasma involves complete hydrolysis followed by quantification of total COL. CMS concentration is then determined as the difference between colistin concentrations measured after and before hydrolysis [18]. Sample preparation typically includes acid hydrolysis, followed by neutralization with NaOH and serum deproteinization. Extraction of the analyte often requires solid-phase extraction, and analysis is generally performed using high-performance liquid chromatography coupled with tandem mass spectrometry (HPLC–MS/MS) [11,19,20,21].

An alternative approach is offered by immunochemical methods, which are simple to perform, do not require expensive equipment, and are routinely applied in laboratory practice for TDM of some antibiotics [22]. Until recently, no reports describing immunoassay-based TDM for CMS were available in the literature apart from one novel biosensor-based approach [23]. This study presents the first reported enzyme-linked immunosorbent assay (ELISA) for COL monitoring in human sera and its application in critically ill patients with burns and pneumonia.

## 2. Results

**Figure 2:**
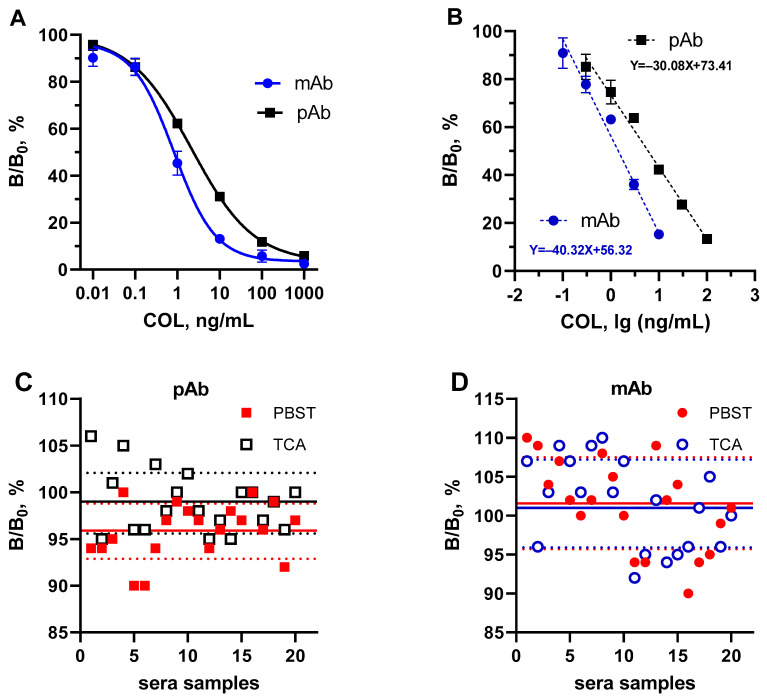
Colistin quantification in pAb-ELISA and mAb-ELISA, and assessment of patients’ sera interferences. (**A**) presents the calibration curve of relative antibody binding (B/B_0_) versus COL concentration; (**B**) shows the linear calibration ranges. (**C**,**D**) illustrate the interference of patients’ sera on polyclonal and monoclonal antibody binding, respectively. B/B_0_—relative antibody binding; COL—colistin; mAb—monoclonal antibody; pAb—polyclonal antibody; PBST—dilution sera samples with assay buffer by 400-fold; TCA—deproteinization of sera samples with 5% trichloroacetic acid and subsequent dilution with PBST at the same factor dilution.

A direct competitive ELISA for COL quantification was developed using previously described anti-polymyxin polyclonal (pAb) and monoclonal (mAb) antibodies [24,25]. Antibodies immobilized in the microplate wells interacted with the PMB-HRP conjugate, while COL in the sample led to concentration-dependent inhibition of this binding, which was quantified using standard calibration curve (Figure 2A,B).

The assay IC_50_ towards COL, CMS and PMB was 2.39, 88.52, and 2.27 ng/mL with pAb and 0.83, 237.14, and 1.68 ng/mL with mAb, respectively (Table 1). The limit of detection for COL was 0.053 ng/mL with pAb-based system and 0.047 ng/mL with the mAb-based system.

Sera samples from critically ill patients not exposed to polymyxins were treated with 5% TCA or simply diluted with PBST, followed by ELISA testing. As illustrated in Figure 2C,D, both pretreatment approaches result in negligible matrix effects, as evidenced by the absence of significant B/B_0_ deviation from 100% antibody binding. Thus, the analysis revealed no significant influence from blood biochemical markers such as albumin (22–43 g/L), fibrinogen (1.1–8.1 g/L), C-reactive protein (0–138 mg/L), bilirubin (5–15.4 mmol/L), creatinine (30–166 µmol/L), and urea (3–18 mmol/L) with indicated variations in applied samples. Similarly, co-administered pharmaceuticals at their peak concentrations, including tigecycline (2.0 mg/L) [26], linezolid (20 mg/L) [27], vancomycin (40 mg/L) [28], amphothericin B (4.3 mg/L) [29], and amikacin (220 mg/L) (unpublished), did not demonstrate a substantial influence on the proposed assay. Thus, the subsequent experiments employed COL calibration in PBST, which coincided with calibration derived from TCA-treated human sera.

Total amount of COL formed after complete hydrolysis of CMS was measured in parallel using two analytical systems in serum samples from healthy volunteers, applying a spike-and-recovery approach. Both systems demonstrated that within the concentration range of 1000–3000–10,000 ng/mL COL recovery from human serum following deproteinization with 5% TCA was within acceptable limits of 80–106% (Table 2).

To ensure complete hydrolysis of CMS in serum samples from healthy volunteers, acid treatment with 5% TCA was applied, thereby achieving both hydrolysis and deproteinization of the samples. CMS hydrolysis was evaluated over incubation periods of 0, 1, and 20 h at 37 °C. Simple dilution of samples in assay buffer was used as a control. The results of the study demonstrated that the conversion of CMS to COL with TCA 5% at 37 °C plateaued within 1 h (Table 3), yielding 76–82% of COL in the pAb-ELISA and 60–76% in the mAb-ELISA. The extended treatment period (20 h) under the prevailing conditions did not result in an augmentation of COL formation, but even caused a slight decrease due to possible degradation [30].

Consequently, CMS conversion under the selected conditions (5% TCA, 1 h, 37 °C) was validated through the use of HPLC-MS/MS identification of COL A and COL B within CMS and COL standards, as well as in the hydrolyzed CMS (Figure S1). It was determined that conversion of CMS in serum following a hydrolysis procedure reached 78% (Table S2), which was in complete accordance with pAb-ELISA data.

**Figure 3:**
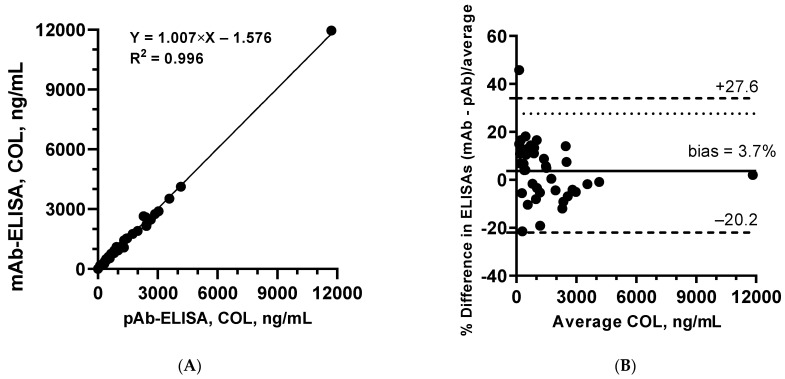
Linear regression between the two methods (**A**) and Bland–Altman plot (**B**) of colistin concentrations in patients’ serum (n = 41) measured with mAb-ELISA and pAb-ELISA. Each value represents the mean (n = 3) ± SD. The 95% limits of agreement are indicated by dashed lines: upper limit (+1.96 × SD), lower limit (−1.96 × SD), and bias.

The suitability of the developed COL immunoassays and the selected sample preparation procedure was evaluated using clinical samples from patients receiving CMS therapy. Parallel analysis of serum samples (n = 41) for COL using two ELISA formats based on immunoreagents (pAb and mAb) with different sub-specificity demonstrated a high degree of correlation between measured COL concentrations (R^2^ = 0.996; Figure 3A). Agreement between the two alternative assays was further assessed using Bland–Altman analysis (Figure 3B), which showed minimal bias (3.7%), with all values falling within the 95% limits of agreement except for one.

To demonstrate the applicability of the developed method, it was applied to describe CMS/COL serum concentrations in critically ill patients receiving CMS therapy. Demographic and clinical characteristics of the patients are presented in Table 4.

**Figure 4:**
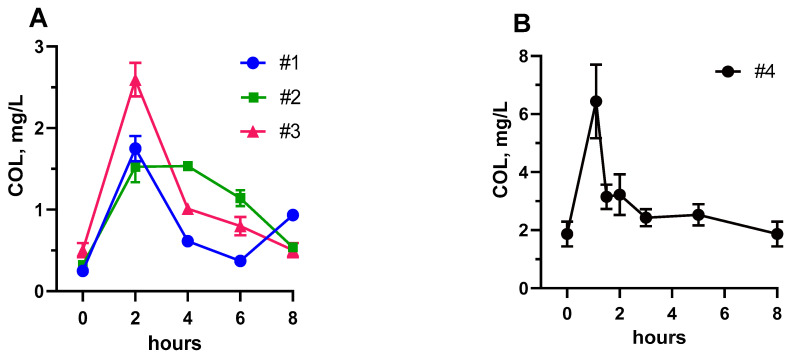
COL concentrations in patients with burns (**A**) and patient with pneumonia (**B**). Colistimethate sodium dose was 80 mg for the patients 1 and 2, 160 mg for patients 3 and 4.

COL concentrations over time in patients receiving CMS are presented in Figure 4. COL concentrations were measured with and without TCA pretreatment indicating no residual CMS in the samples. Based on the total drug concentrations NCA volume of distribution at steady state (Vss) ranged from 41.1 to 64.9 L, while clearance ranged from 7.1 to 16.3 L/h (Table 5).

## 3. Discussion

Polymyxins remain a valuable asset in the treatment of carbapenem-resistant Gram-negative bacteria [16]; however, they are characterized by a relatively narrow therapeutic window (AUC_0–24_ 50–100 mg × h/L) and high incidence of dose-dependent nephrotoxicity [31] and neurotoxicity [32,33]. Importantly, even with the recommended CMS doses of 9 million IU (720 mg of CMS) followed by 4–4.5 million IU (320–360 mg of CMS) every 12 h [16], over 60% of patients with preserved renal function do not achieve the target exposure [34,35]. Using higher doses without TDM is not endorsed by the guidelines.

This indicates TDM might play an important role in CMS therapy optimization; however, its wide implementation has several important obstacles. Most of the modern COL PK studies [15] utilized LC-MS technology, not available routinely in the majority of hospitals.

Alternative immunoanalytical methods could be a good option for measuring COL in blood at the point of care, which would make this test more widely available. The ELISAs of the present study were developed based on antibodies generated against the polymyxin molecule (pAb) or its fragment (mAb). Consequently, both antibodies failed to recognize CMS with blocked amines (Table 1); they could only recognize COL, the same molecule, with free amines. The high cross-reactivity of PMB (105%) does not pose analytical problems, since co-administration of COL/CMS and PMB in clinical practice is not permissible. Conversely, this enables the assay to be used universally for detecting both antibiotics [36].

The newly developed ELISA for quantifying COL in human serum is only the second reported immunoassay applied to TDM of COL in critically ill patients. The previously described biosensor [23] is much more sensitive than the proposed ELISA (LOD of 9 pg/mL vs. 53 pg/mL), but high sensitivity is not required for measuring therapeutic concentrations of colistin (μg/mL), and instead necessitates sample dilution prior to analysis. The biosensor provided a faster result for a single sample compared to ELISA (20 min vs. 90 min). However, it requires the sequential loading of multiple samples for analysis. Therefore, ELISA is characterized by a higher throughput and accessibility than the SPR biosensor.

As CMS is a prodrug, it is essential for therapeutic monitoring to quantify the maximum amount of active drug exposure present in the patient. Sample preparation using 5% TCA simultaneously achieved prodrug hydrolysis and serum deproteinization, standardizing the procedure and minimizing matrix interference. The assay allowed excellent distinction of COL from CMS, as the latter exhibited negligible cross-reactivity (CR 2.7%).

The developed method was applied in a small population of critically ill patients with burns, who often demonstrate significant alterations of drug PK, including increased volume of distribution related to large volume resuscitation and capillary leak, protein catabolism, augmented renal clearance and organ dysfunction [37]. These factors together with the high prevalence of infection in this group require very careful consideration of dosage selection and monitoring of therapy efficacy. To our knowledge, there are at present two studies of COL PK in patients with burns [38,39] which demonstrated increased volume of distribution, edema affecting the CMS to COL turnover and increased clearance of COL proportional to kidney function.

COL concentrations in serum with and without TCA precipitation were similar, indicating CMS instability in vivo and potentially significant ex vivo CMS to COL conversion, including with −20°C storage [40]. Given that total COL concentrations are reported in our study, extra caution is required when interpreting the PK parameters. The study by Corcione et al. [39] in patients with burns also reported total COL concentrations providing valuable reference for our results. In the present study, total drug Vss ranged between 45.7 and 64.9 L in patients with burns, similar to the reference study where the median Vss was 52 L. This is considerably higher than the numbers reported in non-burn populations [15] but is not surprising in the context of severe burns. Total drug clearance was higher in patients with burns, between 8.8 and 16.3 L/h. Both the Vss and CL in the patient without burns were lower.

Significant ex vivo conversion of CMS to COL represents a recognized challenge for TDM implementation in clinical practice, as it introduces potential analytical error regardless of the quantification method used [14,41]. This is recognized by the guidelines, which suggest that C_trough_ might be associated with a lower risk for concentration overestimation, as the CMS concentration in the sample is likely to be negligible given a short half-life [16]. In addition, C_trough_ above 2.6 mg/L is a recognized marker of COL toxicity [42,43,44]. Unfortunately, prediction of AUC from a single C_trough_ is not very accurate [45], making overall assessment of efficacy challenging.

This study has several important limitations which preclude strong clinical conclusions and potentially require further validation steps before the test can be implemented as a monitoring tool. First, the CMS to COL conversion with TCA pretreatment did not achieve complete hydrolysis; however, it was stable and predictable reaching ~80%. Next, the clinical sample size included only four patients which does not allow population PK analysis. Finally, some degree of ex vivo CMS to COL conversion was possible with the study storage conditions, and the PK parameters were therefore reported for the total drug.

## 4. Materials and Methods

### 4.1. Chemicals and Reagents

This study used colistin sulfate BioChemica (MW 1365.64 g/mol) and polymyxin B sulfate (MW 1385.63 g/mol) from AppliChem (Darmstadt, Germany); colistin sodium methanesulfonate BioChemica (MW 1759.9 g/mol) from Sigma (Buchs, Switzerland); rabbit polyclonal antibody (pAb) against whole polymyxin molecule [24] and mouse monoclonal antibody (mAb) 5B10 against polymyxin fragment [25]; polymyxin B–horseradish peroxidase (PMB-HRP) conjugate [36]; trichloroacetic acid (TCA) and 3,3′,5,5′- tetramethylbenzidine (TMB) from Sigma-Aldrich (St. Louis, MO, USA); and solutions for analysis: 0.05 M carbonate–bicarbonate buffer (pH 9.6), 0.15 M phosphate-buffered saline (pH 7.2) with 0.05% Tween-20 (PBS-T).

### 4.2. Patients

Serum samples from healthy volunteers and patients were obtained from the Treatment and Rehabilitation Center Ministry of Health, Moscow, Russia. The study protocol was approved by the local ethics committee (N9 ЭK/061, 22 January 2025). CMS was dosed at the discretion of the attending physician and was administered intravenously over 1 h three times daily: two patients received 240 mg/day and two patients 480 mg/day. Blood samples were collected after at least 48 h of antibiotic therapy, which was assumed to be sufficient to reach steady state.

### 4.3. Determination of Colistin

CMS concentrations in samples were assessed indirectly based on the difference between COL concentrations measured by ELISA with or without hydrolysis. The concentration of COL after complete hydrolysis equaled 0.71 times the respective CMS concentration based on the molecular weight ratio [18].

### 4.4. Hydrolysis of CMS in Human Serum

CMS was spiked in the healthy volunteers’ serum at concentrations of 10,000, 3000, and 1000 ng/mL and incubated for 1 h at 37 °C to simulate in vivo conditions. To replicate the clinical workflow, samples were frozen at −20 °C prior to analysis. Sample preparation involved simple dilution or deproteinization and hydrolysis using 5% TCA at 37 °C, a process designed to eliminate potential matrix effects and convert CMS to COL. To ensure the assay’s measurement range, sample dilution factors of 100–1000 were utilized.

### 4.5. Enzyme-Linked Immunosorbent Assay (ELISA) for COL

The assay was performed using a direct competitive ELISA format. Antibody solutions prepared in carbonate–bicarbonate buffer were added to microplate wells for immobilization. After incubation for 16 h at 4 °C and subsequent washing, antibiotic standards (1000–0.01 ng/mL and 0 ng/mL) prepared in PBS-T and/or appropriately diluted samples were added together with a PMB-HRP conjugate. Plates were incubated in a thermoshaker ST-3 L (ELMI Ltd., Riga, Latvia) for 1 h at 25 °C, washed, and then incubated with a TMB substrate solution. After 30 min the enzymatic reaction was stopped by adding 0.5 M sulfuric acid, and absorbance was measured at 450 nm using a microplate reader LisaScan (Erba Mannheim, Brno, Czech Republic). Antibody binding in wells containing zero analyte concentration (B_0_) was defined as 100%. Relative binding (B/B_0_) was calculated for each analyte concentration.

The working range of the assay was defined as the IC_20_–IC_80_ interval and represented the linear dependence of concentration on B/B_0_ used for quantification of COL. The limit of detection (LOD) was determined as the analyte concentration corresponding to B_0_ − 3 × SD. Specificity was assessed with antibody cross-reactivity calculated as the percentage ratio of the IC_50_ of the primary analyte to that of a structurally related compound:

Cross-reactivity (%) = 100 × IC_50_ (analyte)/IC_50_ (analogue).

Sample preparation and extraction efficiency were evaluated in recovery experiments using sera from healthy volunteers and defined as:

Recovery (%) = 100 × (C_measured_ − C_0_)/C_spiked_,

where C_0_ represents the initial analyte concentration in the sample. Each sample was measured in several replicates. The recovery rate reflected the accuracy of the assay, while the standard deviation (SD) for n = 3 demonstrated its precision.

The possible matrix effect of sera ingredients and co-administered drugs on specific antibody binding was assessed by using B/B_0_ values in pre-treated samples. This material from previous studies includes a panel of sera from critically ill patients not exposed to polymyxins.

### 4.6. High-Performance Liquid Chromatography with Tandem Mass Spectrometry (HPLC–MS/MS)

LC-MS/MS was previously conducted to verify the accuracy of the ELISA measurement [26]. In the present study, the technique was employed to verify the conversion of CMS to COL as a result of the hydrolysis procedure (Section 4.4). A comprehensive description of the HPLC-MS/MS procedure, along with the ensuing results, is provided in the Supplementary Materials.

### 4.7. Statistical and Pharmacokinetic Analysis

Statistical analysis and graph preparation were performed using Excel 2019 (Microsoft Corporation, Redmond, WA, USA) and Prism 9 (GraphPad Software, LCC, Boston, MA, USA). Pharmacokinetic parameters were calculated using the NCA module in MonolixSuite 2024R1 (Lixoft SAS, Antony, France). The area under the concentration–time curve over 8 h (AUC_0–8_) was calculated using sampling at 0, 2, 4, 6, and 8 h.

## 5. Conclusions

This proof-of-concept study presents the first ELISA-based method for TDM of COL applied in critically ill patients receiving CMS therapy. Analytical performance was confirmed by cross-validation of two independent ELISAs based on different recognition elements (pAb and mAb), demonstrating excellent inter-assay agreement (R^2^ = 0.996, bias 3.7%), and the clinical data suggest augmented COL clearance and a risk of subtherapeutic exposure in patients with burns. Limitations of this study, including absence of direct LC-MS/MS comparison, recognized ex vivo CMS to COL conversion, and small sample size, should be addressed in future studies to advance the developed method towards broader clinical application. Further development of the methodology may allow a scalable framework for fast and accessible COL TDM, which together with dedicated popPK studies would lead to more individualized dosing in the most vulnerable patient populations.

## Figures and Tables

**Table 1:** ELISA sensitivity and cross-reactivity.

Analyte	pAb-ELISA				mAb-ELISA			
	IC_50_, ng/mL	CR, %	IC_20_–IC_80_, ng/mL	LOD, ng/mL	IC_50_, ng/mL	CR, %	IC_20_–IC_80_, ng/mL	LOD, ng/mL
COL	2.39	100	0.22–9.95	0.053	0.83	100	0.15–4.68	0.047
CMS	88.52	2.7			237.14	0.35		
PMB	2.27	105.3			1.68	49.3		

CR—cross reactivity (%); CMS—colistimethate sodium; COL—colistin; IC_20_, IC_50_, IC_80_—analyte concentrations resulting in 20, 50 or 80% inhibition of antibody binding, respectively; LOD—limit of detection.

**Table 2:** Recovery of colistin from serum samples of healthy volunteers.

Spiked COL, ng/mL	Recovery (%), Mean ± SD			
	pAb-ELISA		mAb-ELISA	
	PBST, %	TCA, %	PBST, %	TCA, %
10,000	82.1 ± 5.1	84.0 ± 8.2	81.6 ± 11.7	90.4 ± 6.5
3000	99.4 ± 10.3	95.1 ± 10.6	71.8 ± 5.6	80.2 ± 3.8
1000	105.5 ± 4.2	106.2 ± 4.5	96.6 ± 6.9	106.2 ± 5.0

COL—colistin; mAb—monoclonal antibody; pAb—polyclonal antibody; PBST—phosphate-buffered saline with Tween-20; TCA—trichloroacetic acid.

**Table 3:** CMS to COL conversion (%) in serum samples from healthy volunteers under different conditions.

Spiked CMS, ng/mL	COL Equivalent, ng/mL	CMS to COL Conversion (%), Mean ± SD			
		Sample Pretreatment			
		PBST	TCA		
		0 h, 37 °C	0 h, 37 °C	1 h, 37 °C	20 h, 37 °C
pAb-ELISA					
10,000	7100	23.0 ± 4.4	48.4 ± 4.0	76.4 ± 4.1	60.6 ± 10.1
3000	2130	20.5 ± 4.7	46.8 ± 7.3	79.0 ± 0.6	77.0 ± 8.3
1000	710	32.8 ± 5.6	71.4 ± 9.9	82.3 ± 1.6	77.7 ± 10.7
**mAb-ELISA**					
10,000	7100	2.5 ± 0.4	43.5 ± 2.6	60.0 ± 6.7	53.9 ± 5.5
3000	2130	1.3 ± 0.3	66.1 ± 2.1	76.1 ± 1.0	67.0 ± 3.9
1000	710	0.3 ± 0.1	58.1 ± 3.2	65.0 ± 3.3	59.6 ± 5.8

COL—colistin; mAb—monoclonal antibodies; pAb—polyclonal antibodies; PBST—phosphate-buffered saline with Tween-20; TCA—trichloroacetic acid.

**Table 4:** Clinical and demographic characteristics of the study population.

Patient	№ 1	№ 2	№ 3	№ 4
Age, years	31	39	40	87
Weight, kg	55	75	55	70
BMI, kg/m^2^	19	23.7	20.4	26.7
Burn, TBSA%	33/21	45/29	38/23	-
Creatinine clearance, mL/min *	97	155	107	29
Creatinine, μmol/L	76.3	59.6	63.3	155
CMS dose, mg/day	240	240	480	480

BMI—body mass index; CMS—colistimethate sodium; TBSA—total burn surface area. * Creatinine clearance calculated by Cockroft-Gault formula.

**Table 5:** COL pharmacokinetics in critically ill patients.

Patients	№ 1	№ 2	№ 3	№ 4
Dose CMS, mg/8 h	80	80	160	160
AUC_0–24 h_, mg × h/L	19.98	27.78	29.46	67.38
C_max_, mg/L	1.75	1.53	2.59	6.44
C_last_, mg/L	0.94	0.54	0.5	1.87
CL, L/h	12	8.81	16.3	7.12
Vss, L	45.7	56.4	64.85	41.11

AUC_0–24 h_—area under the concentration-time curve over 24 h; CL—clearance; C_last_—last observed concentration (pre-dose); C_max_—maximum concentration; V_ss_—volume of distribution at steady state.

## Data Availability

The original contributions presented in the study are included in the article and Supplementary Materials, further inquiries can be directed to the corresponding authors.

## Supplementary Materials

The following supporting information can be downloaded at: https://www.mdpi.com/article/10.3390/ph19060880/s1, HPLC–MS/MS analysis; Table S1: MRM parameters for COL A and COL B; Figure S1: HPLS-MS/MS chromatograms of CMS and COL in water and human serum before and after hydrolysis procedure; Table S2: Conversion of colistimethate (CMS) to colistin (COL) based on HPLC-MS/MS calculations.
