# Supplementary material for: Immunoassay for Colistin Monitoring in Critically Ill Patients Receiving Colistin Methanesulfonate Therapy
Source: Pharmaceuticals (Basel). 2026 Jun 1;19(6):880. doi: 10.3390/ph19060880 (PMC13304614; doi:10.3390/ph19060880)
Supplement: Supplementary file 1 [file pharmaceuticals-19-00880-s001.zip › pharmaceuticals-4270911-supplementary.pdf]

pharmaceuticals

Supplementary information

Immunoassay for Colistin Monitoring in Critically Ill Patients Receiving Colistin Methanesulfonate Therapy

Yury A. Surovoy [1,2,*], Inna A. Galvidis [3], Akmal I. Alimov [3,4,5], Zhanhui Wang [6], Artem O. Melekhin [7] and Maksim A. Burkin [3,*]

1 Department of Critical Care, University College of London Hospital, London NW1 2BU, UK
2 Faculty of Science and Technology, Middlesex University, London NW4 4BT, UK
3 Department of Immunology, I. Mechnikov Research Institute for Vaccines and Sera, Moscow 105064, Russia;
galvidis@yandex.ru (I.A.G.); aai.alimov@gmail.com (A.I.A.)
4 Faculty of Medicine, M.V. Lomonosov Moscow State University, Moscow 119991, Russia
5 Federal Center for Treatment and Rehabilitation, Ministry of Health, Moscow 125367, Russia
6 College of Veterinary Medicine, China Agricultural University, Beijing 100193, China;
wangzhanhui@cau.edu.cn (Z.W.)
7 Federal Center for Animal Health,Vladimir, 600901 , Russia; artem150196@mail.ru (A.O.M.)
* Correspondence: yury.surovoy@nhs.net (Y.A.S.); burma68@yandex.ru (M.A.B.)

HPLC–MS/MS analysis

An ExionLC system equipped with a binary pump and an autosampler was used for chromatographic separation, which was carried out using an Exlipce Plus C18 column (2.1 mm × 150 mm, 5 µm). The column oven and the autosampler were maintained at 40 and 15 ◦C during operation, respectively. The analytes were separated with a mobile phase consisting of a combination of 0.5% FA acid in water (eluent A) and 0.5% FA in ACN:MeOH (50:50, v/v) (eluent B). The gradient program was as follows: 0–0.5 min: 5 % B; 0.5–4 min: increase from 5% to 100% B; 4–5 min: 100 % B; 5–5.5 min: return to 5% B; 5.5–6.5 min: 5% B. Mobile phase flow rate was 0.3 mL min− 1 . The injection volume was 10 µL. A triple quadrupole mass spectrometer SCIEX Triple Quad™ 5500 (AB Sciex, Singapore) was configured to collect data in the multiple reaction monitoring (MRM) mode. The following optimal parameters were set: voltage on the spraying capillary – 4500 V; injector temperature – 550 ◦C; nitrogen was used as the curtain gas and the collision gas; collision gas pressure – 10 psi; curtain gas pressure – 35 psi; drying and spraying gas pressure – 50 psi; input potential – 10 V. Multiple reaction monitoring (MRM) conditions, declustering potential (DP) and collision energy (CE) were first optimized for COL A and COL B by infusing solutions standards prepared in the mobile phase. Characteristic molecular ions were selected as precursor ions, and two product ions were monitored for COL A and COL B. MRM parameters and retention times are provided in Table S1.

Table S1. MRM parameters for COL A and COL B

Analyte	Precursor ion	Precursor Ion (m/z)	Product Ion (m/z)	Collision Energy (eV)	Retention Time (min)
Colistin A	$[M+2H]^{2+}$	585.5	101.0	24	3.3
	$[M+2H]^{2+}$	585.5	241.1	21	3.3
Colistin B	$[M+2H]^{2+}$	578.8	101.0	25	3.3
	$[M+2H]^{2+}$	578.8	227.1	22	3.3

CMS is not a single chemical compound, but rather a complex multicomponent mixture of partial sulfomethylation products formed during the manufacturing process. Due to the fact that CMS exhibits a lack of strong UV absorption and heterogeneity, the majority of validated methods rely on the chemical conversion of CMS to COL, followed by detection. Consequently, an indirect approach was employed, entailing complete hydrolysis of CMS and subsequent quantitative determination of the resulting COL.

In order to perform an objective assessment of hydrolysis under the selected conditions (5% TCA, 1 hour, 37°C), a series of comparative chromatographic analyses were conducted. The efficiency of CMS hydrolysis in serum was assessed by comparison with corresponding colistin A and B standards subjected to the same sample preparation procedure. A subsequent analysis entailed a comparison of HPLC-MS/MS chromatograms (Figure S1).

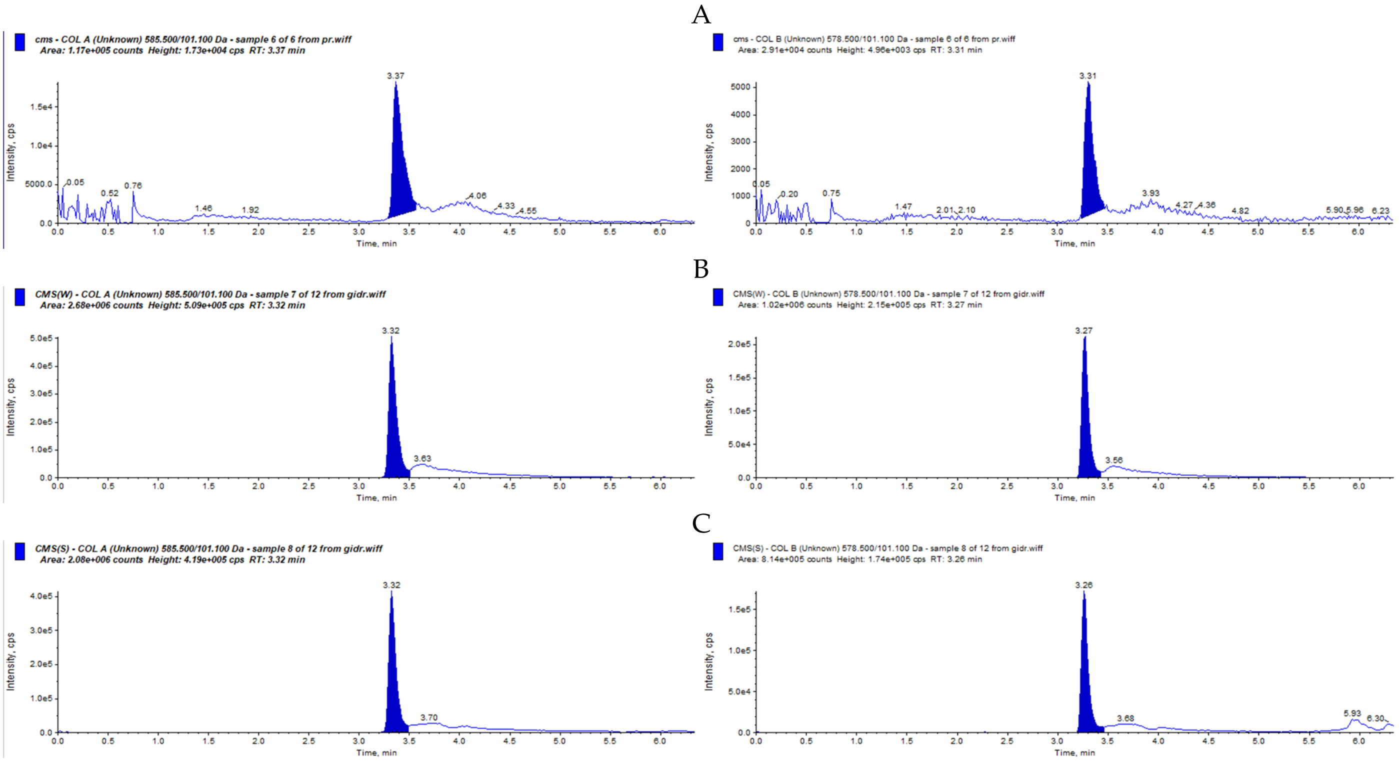

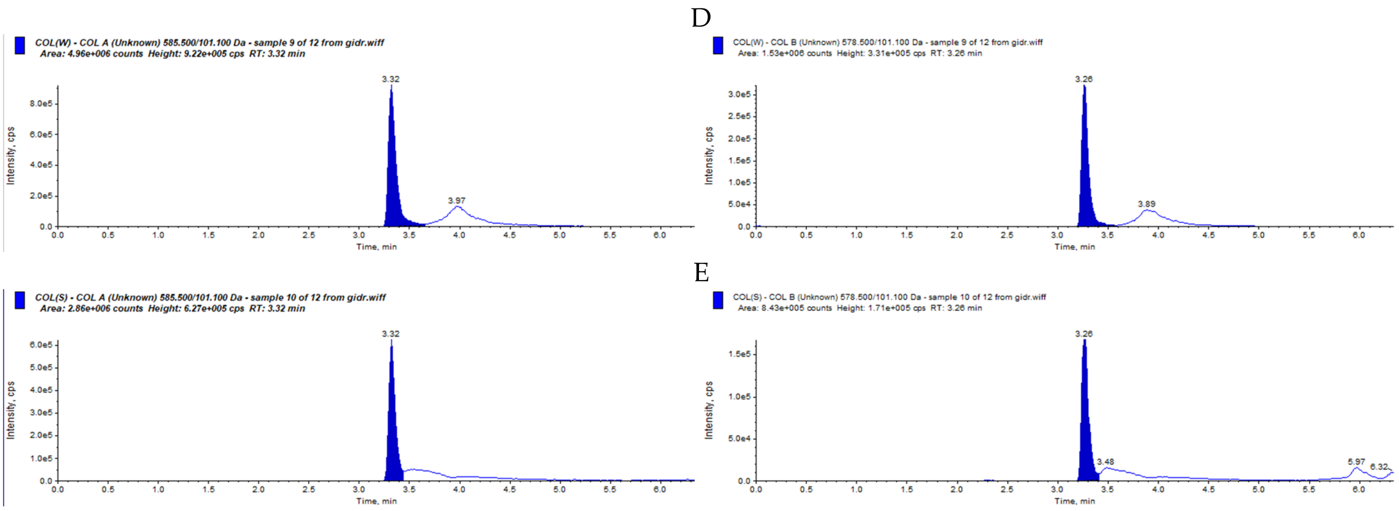

Figure S1. HPLS-MS/MS chromatograms of CMS and COL in water and human serum before and after hydrolysis procedure.

A. CMS solution; B. CMS hydrolyzed in aqueous solution; C. CMS hydrolyzed in normal human serum; D. COL standard in aqueous solution; E. COL standard in normal human serum. The left panel of the figure presents the identification of COL A, while the right panel displays the chromatograms for COL B.

Table S2. Conversion of colistimethate (CMS) to colistin (COL) based on HPLC-MS/MS calculations.

Figure S1	Analyte	Area under peak, cps			Conversion of CMS to COL, %
		COL A	COL B	COL A +B*	
A	CMS standard (W)	1,17E+05	2.91E+04	1,46E+05	2,25
B	CMS hydrolyzed (W)	2,68E+06	1,02E+06	3,70E+06	57,01
C	CMS hydrolyzed (S)	2,08E+06	8,14E+05	2,89E+06	78,15
D	COL standard (W)	4,96E+06	1,53E+06	6,49E+06	100
E	COL standard (S)	2,86E+06	8,43E+05	3,70E+06	100

*The calculation is predicated on the assumption that the calibration for COL A and COL B will be approximately the same; therefore, the sum of the areas is simply calculated. W-water; S – human serum;
